# Numerical Simulation of Temperature Variations during the Application of Safety Protocols in Magnetic Particle Hyperthermia

**DOI:** 10.3390/nano12030554

**Published:** 2022-02-06

**Authors:** Gerasimos Pefanis, Nikolaos Maniotis, Aikaterini-Rafailia Tsiapla, Antonios Makridis, Theodoros Samaras, Mavroeidis Angelakeris

**Affiliations:** 1 Faculty of Sciences, School of Physics, Aristotle University, 54124 Thessaloniki, Greece; gpefanis98@gmail.com (G.P.); aitsiapl@physics.auth.gr (A.-R.T.); anmakrid@physics.auth.gr (A.M.); theosama@physics.auth.gr (T.S.); agelaker@auth.gr (M.A.); 2 MagnaCharta, Center for Interdisciplinary Research and Innovation (CIRI-AUTH), 57001 Thessaloniki, Greece

**Keywords:** magnetic particle hyperthermia, magnetic nanoparticles, eddy currents reduction, tissue sparing

## Abstract

Unavoidably, magnetic particle hyperthermia is limited by the unwanted heating of the neighboring healthy tissues, due to the generation of eddy currents. Eddy currents naturally occur, due to the applied alternating magnetic field, which is used to excite the nanoparticles in the tumor and, therefore, restrict treatment efficiency in clinical application. In this work, we present two simply applicable methods for reducing the heating of healthy tissues by simultaneously keeping the heating of cancer tissue, due to magnetic nanoparticles, at an adequate level. The first method involves moving the induction coil relative to the phantom tissue during the exposure. More specifically, the coil is moving symmetrically—left and right relative to the specimen—in a bidirectional fashion. In this case, the impact of the maximum distance (2–8 cm) between the coil and the phantom is investigated. In the second method, the magnetic field is applied intermittently (in an ON/OFF pulsed mode), instead of the continuous field mode usually employed. The parameters of the intermittent field mode, such as the time intervals (ON time and OFF time) and field amplitude, are optimized based on the numerical assessment of temperature increase in healthy tissue and cancer tissue phantoms. Different ON and OFF times were tested in the range of 25–100 s and 50–200 s, respectively, and under variable field amplitudes (45–70 mT). In all the protocols studied here, the main goal is to generate inside the cancer tissue phantom the maximum temperature increase, possible (preferably within the magnetic hyperthermia window of 4–8 °C), while restricting the temperature increase in the healthy tissue phantom to below 4 °C, signifying eddy current mitigation.

## 1. Introduction

Magnetic particle hyperthermia (MPH) is a cancer treatment modality that exploits the interactions of magnetic fields with magnetic nanoparticles (MNPs) to generate heat, predominantly via magnetic hysteresis loss, in order to increase the tumor temperature in the range of 41–45 °C [1,2,3,4]. Since cancer cells are more susceptible than healthy ones to 41–45 °C, a rise within this temperature region leads to the suppression of cancer cells growth and tumor shrinkage [5,6]. MPH received regulatory approval by the European Medicines Agency to treat recurrent glioblastoma in combination with radiotherapy in 2010 [7] and Investigational Device Exemption (IDE) approval from the U.S. Food and Drug Administration (FDA) in 2018 to conduct prostate cancer clinical trials [8,9]. The tumoral region containing the magnetic nanoparticles, which are usually made of iron oxide and dispersed in aqueous solution, is exposed to an alternating magnetic field (AMF) [10]. Low frequency (<1 MHz) AMFs are essentially not attenuated by tissue and thus penetrate deep into the body. Repeated MPH treatment after a single MNPs injection has been clinically demonstrated for prostate and brain tumors [11,12,13]. The stable colloidal dispersion of magnetic nanoparticles, usually called ferrofluid, is injected directly or delivered to the tumor via passive or active (functionalized) targeting upon intravenous administration [14,15]. Thus, MPH offers the potential for the well-controlled and repeated heating of deep tissues by controlling AMF power to modulate the heat sources embedded in the tumor [16]. AMF coils generating homogeneous AMF in the target region, reduce nonspecific tissue heating compared to currently available clinical MPH systems by reducing the requirement for increased amplitude at the surface to compensate for the reduced amplitude at the target [17].

However, when considering clinically relevant volumes of tissue, one of the factors which most significantly limits treatment efficacy is the adverse effect of the non-specific heating, due to eddy currents (EC) in healthy (non-MNPs bearing) tissue. Interactions of AMF with electrically conducting (diamagnetic) bodies, such as human tissues, induce EC, which deposit Joule heat to tissues [18]. The nonspecific eddy current heating depends on the induced electric field amplitude and the electrical conductivity of the tissue. The clinically permissible limit for a 30 cm diameter region of tissue, is [19] H × f < 4.85 × 10^8^ A/m∙s, where H and f are the magnetic field amplitude and frequency, respectively. For a successful MPH treatment, magnetic hysteresis loss power deposited by the MNPs should always be higher than non-specific heating, due to EC. EC heating can be reduced by limiting the area of exposure to high-amplitude AMF, as well as by reducing the amplitude or application mode [20,21,22]. Pulsed AMF—that is, reduced Duty Cycle—dissipates heat generated by eddy currents as demonstrated in literature. In Tsiapla et al. [23] the role of an intermittently applied AMF was evaluated on the thermal response of iron oxide (Fe_3_O_4_) MNPs dispersed in phantom and ex vivo samples with respect to field parameters and in direct comparison with the corresponding continuously applied AMF. The parameters of the intermittent field mode, such as time intervals (ON time: 25–100 s, OFF time: 50–200 s, Duty Cycle: 16–100%) and field amplitude (30–70 mT) were optimized based on computations with healthy tissue and cancer tissue phantoms. Moreover, Kumar et al. [20] had shown that pulsed AMF enabled physiological thermoregulatory processes as examined, in vivo, in a mouse model exposed to high-amplitude AMF.

The constant motion of the coil relative to the tumor has also been proven to be an adequate strategy for attenuating the EC heating. Stigliano et al. [24] presented a coil technique for moving MNPs containing tissue phantom in order to manipulate the tissue exposed to the field and to decrease the thermal dose, due to EC heating by considering the placement of the tissue in time and space, relative to the AMF—or vice versa. Although a decrease in the maximum heat deposition in non-cancerous regions was achieved by the authors, there are some limiting factors concerning the implementation of this technique. The first one is the erosion of the sample phantom, which is often required to be replaced in experiments. The second one is the complexity of the Pennes bioheat equation [25] that needs to be solved for the computational evaluation of the treatment protocols. To overcome this difficulty, Neufeld et al. [26] developed an approximation approach to predict the temperature increase during magnetic resonance imaging (MRI) radiofrequency exposure. The approximation assumes that the temperature increase exhibits exponential behavior and eventually tends to equilibrium. This technique was adopted in a numerical study of our previous work by Balousis et al. [27], where temperature increase was estimated in two protocols that involved the simulation of coil displacement, relative to a tumor-bearing phantom tissue during the exposure. In the first protocol, the linear motion of the coil on one side with respect to the hypothesized tumor location inside the phantom was simulated. The estimated maximum temperature increase in the healthy tissue and tumor was reduced by 12 and 9%, respectively, compared to a stationary coil. The second technique involved a symmetrical variation of the first one, where the coil was moving left and right of the phantom in a bidirectional fashion. This protocol was considered as the optimum one, since the estimated maximum temperature rise of the healthy tissue and tumor was reduced by 25 and 1%, respectively, compared to the control protocol.

In this work, two alternative protocols of magnetic hyperthermia will be numerically studied to improve the treatment. The first goal is to reduce the eddy currents, and therefore the heating due to them, to the healthy tissues by avoiding overheating during the treatment. The second goal is to maintain the temperature of the cancer tissues at the desired levels, while simultaneously protecting the healthy tissues. The two alternative protocols studied are: (i) the simulation of a moving magnetic field source instead of the stationary one typically used. In order to apply the method developed by Balousis et al. [27] in less mild conditions of MPH (higher H × f) and to investigate the factors that affect it, the motion of the coil in relation to a non-MNPs-bearing (first sample under study) and MNPs-bearing phantom tissue (second sample under study) was investigated. Henceforth, the first sample will be denoted as healthy tissue phantom (HTP), while the second will be cancer tissue phantom (CTP). The coil was set in motion sequentially below the phantoms, which were elevated to a fixed position above it. The motion of the coil was a sinusoidal linear motion centered on its initial position, i.e., the position just above the coil. (ii) The simulation of an intermittently applied alternating magnetic field, in a pulse mode, instead of the constant alternating magnetic field used in classical hyperthermia. A well-elaborated numerical approach provided a rapid calculation of the temperature increase and, furthermore, the ability to quickly simulate a variety of Duty Cycles, operational times, ON/OFF sequences, and field amplitudes. Thus, this model provides the optimum conditions that satisfy the two aforementioned goals of this work and, consequently, it may also save experimental time.

The implementation of these two methods was conducted using two computational models constructed to predict the temperature behavior of phantom tissues. Hereafter, the first technique will be referred to as moving coil (MC) hyperthermia and the second as pulsed magnetic field (PMF) hyperthermia. Additionally, the reliability of the computational models was evaluated after their comparison to the experimental results. 

In general, the ability to quickly simulate a large number of different protocols is a sine qua non prerequisite for translation of the presented technique into clinical practice in the future. In this way, someone can use this approach either before or after experimental measurements to quickly optimize the treatment protocol parameters of either the MC or the PMF method.

## 2. Materials and Methods

### 2.1. Selection of MC Hyperthermia Settings and Exposure Protocol

**Figure 1:**
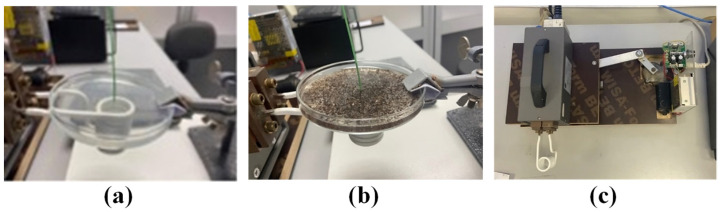
Experimental setup for measuring temperature rise in HTP (**a**), CTP (**b**), and Coil drive mechanism (**c**).

The MC hyperthermia method involves moving the coil relative to the phantom tissue during the exposure. As shown in Figure 1a,b, the bottom surface of each phantom (placed in a large Petri dish) was parallel to the surface defined by the first turn of the coil and placed 1 mm above it. The optical fiber that measures the temperature was positioned at the center of the phantom and 1 mm above the bottom surface. The optical fiber measurements were recorded on a computer using appropriate software. For both HTP and CTP samples, the experiment was performed sequentially under conditions of applied current amplitude and frequency of 160 A and 375 kHz, respectively. For the MC hyperthermia experiment, the coil moved below the samples by applying a servomechanism, as shown in Figure 1c. The motion of the coil was a linear oscillation around the center of the phantoms. More specifically, the coil is moving symmetrically—left and right—relative to the specimen and in a bidirectional fashion. The time of hyperthermia exposure was 900 s for both HTP and CTP samples.

A suitable computational model was constructed to evaluate the performance of this method by testing motions with different maximum distances. The experimental data used to further validate our method were those obtained in two cases: coil (i) movement with a maximum distance of 8 cm and (ii) stationary coil (classical configuration of magnetic hyperthermia).

### 2.2. Selection of PMF Hyperthermia Settings and Exposure Protocol

The two characteristic parameters of PMF are the ON and OFF times, which determine the time, when the field source is switched on (ON) and the time that it is switched off (OFF) and thus, compose the final form of the field pulses. One way to express the ratio between ON and OFF times is the Duty Cycle, shown in Equation (1). The role of pulsed AMF—that is, Duty Cycles—to reducing eddy current heating was based on our previously reported study [23]. This quantity is defined as the percentage of the time the coil is in operation (ON) to the total time of a cycle (ON + OFF).

(1) $$\mathrm{Duty}\;\mathrm{Cycle}=\frac{\mathrm{Field}\;\mathrm{ON}\;\mathrm{time}\;\left(s\right)}{\mathrm{Field}\;\mathrm{ON}\;\mathrm{time}\;\left(s\right)+\;\mathrm{Field}\;\mathrm{OFF}\;\mathrm{time}\;\left(s\right)}\times 100\%$$

In this work, PMF hyperthermia was studied using a coil, whose operation can be adjusted so that the heating source is ON or OFF for a certain period of time. The samples were placed above the coil. Using experimental data from heating and cooling curves that were recorded after conventional MPH (continuous application of AMF), the appropriate numerical model was constructed to investigate PMF hyperthermia. All magnetic hyperthermia experiments were performed with a total time of 900 s. A large number of different ON/OFF combinations and different operating cycles was rapidly evaluated using this model that predicts the temperature behavior of a sample, when it is exposed to an alternating magnetic field. The model also predicts the cooling of the sample in the absence of a field. In this way, multiple parameters for the PMF hyperthermia were tested, saving the time and effort required to perform the respective experiments. Different ON and OFF times were examined in the range of 25–100 s and 50–200 s, respectively, and under various AMF amplitudes (45–70 mT).

### 2.3. Algorithm Description for MC Hyperthermia Simulation

The numerical method used to determine the temperature increase in each sample is based on the approach of Neufeld et al. [18] i.e., that the temperature *T* exponentially approaches a maximum constant value $T_{inc}$ and its temporal evolution *T*(*t*) is given by the Equation (2).

(2) $$Τ\left(t\right)=T_{inc}\left(1-e^{-\frac{t}{\tau }}\right)+{Τ}_{start}$$

In the case of the moving coil, the temperature increase Δ*Τ* with a time step Δ*t_i_* is given by Equation (3). This equation links the increase in temperature to the specific absorption rate (*SAR*) value at each time step.

(3) $$\Delta {Τ}_{i+1}=\Delta {Τ}_{i}+\left(1-e^{-\frac{\Delta t}{\tau }}\right)\left(cSAR_{i}-\Delta {Τ}_{i}\right)$$

where *SAR_i_* is the total heating rate deposited from both eddy currents and the *MNPs* at time *i* to the phantom tissues and given by the following equation:

(4) $$SAR_{i}=SAR_{EC}+SAR_{MNPs}$$

In Equation (3) the constant *c* was taken equal to *c* = 0.38 K∙kg/W [27] and is defined as a constant that depends on the nature of the tissue (anatomy, perfusion, and thermoregulation), while the time constant *τ* was calculated by fitting an experimental *T*(*t*) curve, obtained for a non-moving coil, to Equation (2). Throughout the simulations the *SAR*, due to EC and the *SAR*, due to *MNPs* are given, respectively, by the following equations:

(5) $$SAR_{EC}=\frac{\sigma {\vec{Ε}}^{2}}{2\rho_{ph}}$$

(6) $$SAR_{MNPs}=\frac{c_{MNPs}\left(A\times f\right)}{\rho_{MNPs}}$$

where in the Equation (5), *σ* and $\rho_{ph}$ are the electrical conductivity and the density of the phantom tissue and $\vec{Ε}$ the value of the electric field at the specific point, where the measurement is made. In Equation (6), *A* is the area of the MNPs hysteresis loop, *f* is the frequency of the magnetic field, $c_{MNPs}$ and $\rho_{MNPs}$ are the concentration and the density of MNPs dispersed in the CTP sample, respectively. For the HTP sample only the $SAR_{EC}$ is considered in Equation (4). In the experiment, the coil moves relative to the phantom. In the numerical model, an equivalent approach was taken, where the phantom moves within a spatially changing magnetic and electric field created by the coil.

The motion of the phantom, like the coil in the experiment, is a linear oscillation with a maximum displacement $x_{max}$ given by the following sinusoidal equation:

(7) $$x=x_{max}\sin\left(2\pi f_{MC}t\right)$$

where *f_MC_* is the frequency of oscillation for the moving coil. In the experiment, the coil was set to perform seven full circles during one minute of oscillation and thus *f_MC_* was taken equal to 7/60 Hz in the simulations.

Finally, the temperature increase is calculated using an algorithm in MATLAB R2021a. More specifically, $\Delta {Τ}_{i+1}$ is calculated through Equation (3) at the position, where the optical fiber is placed. The calculation of $S_{i}$ as a function of the spatially varied electric and magnetic field, E(x) and H(x)—in each position—was done by entering in the algorithm for the frequency of motion *f* and the value of $x_{max}$. Then, the position is calculated for each time step from Equation (7) and then the values of the magnetic and electric field are calculated through the approximate mathematical relations E(x) and H(x). The result is a file containing two columns of data, namely time and temperature increase. More details about field calculations can be found in the Supplementary Materials. There, the spatially varied electric and magnetic fields E(x) and H(x) are calculated using COMSOL Multiphysics and their distribution is demonstrated in Figure S3.

### 2.4. Algorithm Description for PMF Hyperthermia Simulation

The numerical method used to determine the temperature rise is based, as in the case of the MC hyperthermia, on the approach of Neufeld et al. [26]. In this case the temperature increase Δ*Τ* with a time step Δ*t* is given by Equation (8).

(8) $$\Delta {Τ}_{i+1}=\Delta T_{i}+\left(1-e^{-\Delta t_{i}/\tau_{j}}\right)\left(T_{inc}\times \kappa -\Delta T_{i}\right)$$

In Equation (8) *κ* is a constant that equals to 1, when the AMF is ON and to 0 when the AMF is OFF, resulting in the local heating and cooling of the phantom, respectively.

By swapping *κ* between 0 and 1, the value of time constant $\tau_{j}$ is also varied between two characteristic values, *τ_c_* and *τ_h_*, which are the exponential constants for cooling and heating of the specific sample under study, respectively.

To approximate the temperature increase Δ*Τ* with Equation (8) in each discrete time step of MPH experiment, an algorithm was utilized with MATLAB. To start with, we performed a fitting function based on Equation (2) to the experimental heating and cooling curves for the case of continuously applied AMF to estimate the maximum temperature $T_{inc}$, for a specific duration of time, as well as for the corresponding time constants *τ_c_* and *τ_h_*. The time step $\Delta t_{i}$ was set to 0.1 s. This method of limited time interval selecting ensures that the fitting parameters are accompanied with a reasonably small standard deviation.

After substituting the estimated $T_{inc}$ and $\tau_{j}$ (equal to *τ_c_* when cooling and *τ_h_* when heating the sample) values in Equation (8), we got the temporal evolution of temperature during the intermittent application of the AMF. The algorithm identifies in which time intervals the coil is operating and in which intervals it is switched off, and, given this, the values of the vector *κ* are set equal to 1 and 0, respectively. Initially, temperature increase $\Delta {Τ}_{0}$ is zero. At each time step, $\Delta T_{i}$ is updated by Equation (8), offering a rapid calculation of $\Delta T_{i}$ and, furthermore, the ability to quickly simulate a large number of different Duty Cycles.

In all the curves, the MPH characteristic window (41–45 °C) in the diagrams is between 4 and 8 °C, since the increase of the temperature Δ*Τ* is studied here and the initial temperature is considered 37 °C.

### 2.5. Magnetic Nanoparticles

The magnetite magnetic nanoparticles used to carry out the experiments presented in the work were fabricated using the method of chemical coprecipitation. Chemical reagents with high purity were purchased from the Merck Chemical Company, Darmstadt, Germany. Optimum synthesis was done by adding 5 g of ferrous sulfate heptahydrate and 7.1 g of ferric sulfate 9-hydrate into 400 mL of distilled water in a ratio 1:2. The reaction was maintained for 30 min at 70 °C under stirring and, for the conversion of excess iron species into Fe_3_O_4_, 2M of NaOH solution was added (~50 mL of NaOH). The precipitate was washed with distilled water (three times) and left to dry at 40 °C. The magnetic characterization of MNPs was achieved with a vibrating sample magnetometer (VSM) at 300 K under a static applied field of 1 T. The saturation magnetization was found equal to 96.3 emu/g. The magnetization versus magnetic field, M(H), dependence is shown in Figure S15 of the Supplementary Materials, where the hysteresis loop was measured at 1 T.

### 2.6. Tissue Phantoms

Biomimetic materials are widely used in literature, due to their biophysical properties [28,29]. Among them, agarose is a perfect candidate that simulates the mechanical and thermoelectric properties of tissues. Two different phantoms based on agarose solutions with and without the presence of MNPs that simulate cancer and healthy tissue, respectively, were synthesized. More specifically, for the HTP, 0.12 g of agarose and 0.12 g of sodium chloride (NaCl) were dissolved in 30 mL of distilled water after temperature adjustment to 85 °C. After they had been completely dissolved, the liquid solution was transferred to a Petri dish (~10 cm) and left at room temperature until it turned into a gel.

The CTP was made in the exact same way, but MNPs (0.12 g) were added and the liquid solution inside a Petri-dish was placed in an ultrasonic bath in order to achieve a uniform distribution of MNPs, as the liquid solution gradually converted into gel. At this point, it should be mentioned that NaCl was used only to increase electrical conductivity so that the increase in temperature, due to eddy currents, was apparent.

### 2.7. AMF System

**Figure 2:**
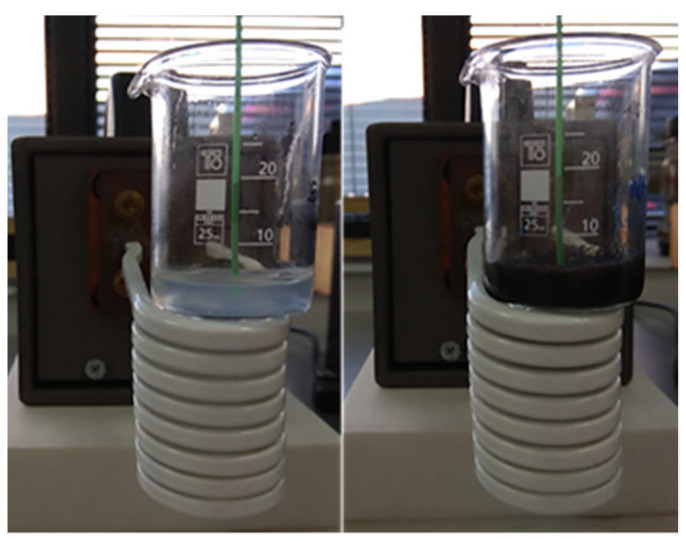
Experimental setup of PMF hyperthermia for HTP (**left**) and CTP (**right**) samples.

The device used to conduct the MPH experiments consisted of a common coil (1.2 kW Ambrell Easyheat 0112, New York, United States), which induces an alternating magnetic field of frequency 375 kHz and amplitude 30–70 mT, according to the applied AC current. The magnetic field calibration for the various AC amplitudes had been achieved in our previous work by employing electromagnetic simulations with a suitable finite elements method (FEM) model of the used experimental setup [23], as shown in Figures S1 and S2 of the Supplementary Materials. As shown in Figure 2, an optical fiber was positioned in the center of the phantom, which is placed in a beaker, and the temperature was measured at this point with a time step of 0.4 s. The measurements were recorded with appropriate software. During the experiment, we assumed that the temperature recorded in the center of the sample was representative of the temperature of the entire sample.

## 3. Results and Discussion

### 3.1. Proof of Principle

**Figure 3:**
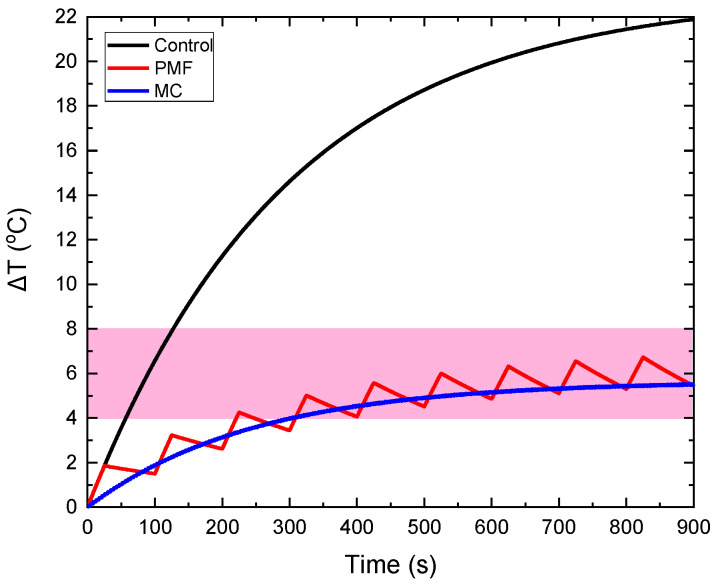
Illustration of the feasibility of our method. The control Δ*T* curve corresponds to the classical MPH method and is compared to the Δ*T* curves obtained from MC and PMF methods under 8 cm maximum coil displacement and 25% Duty Cycle, respectively. The magnetic field at the measuring point of temperature was equal to 60 mT, as shown by the field simulations of Figure S2a in the Supplementary Materials. All Δ*T* curves correspond to the CTP sample.

In Figure 3, a realization of our strategy is depicted in order to demonstrate its feasibility and practical potential. The temperature increase in MNPs-bearing phantom (CTP sample) obtained from classical MPH, denoted as the control curve, is compared to the ones obtained from the MC and PMF hyperthermia methods. The results are given for an applied magnetic field equal to 60 mT. In the PMF case, the Duty Cycle was equal to 25% (ON/OFF = 25/75 s), while in the MC case, the maximum distance was equal to 8 cm. Although Δ*Τ* is decreased, it is retained within the MPH characteristic window (41–45 °C), resulting simultaneously in the sparing of the surrounding healthy tissue, as it will be demonstrated in the next sections, for all the samples and conditions studied here.

In the following results, we study the influence of various parameters, such as the maximum displacement, the Duty Cycle, and the magnetic field amplitude, on the temperature increase in the CTP and HTP samples. The optimum conditions for each case study are determined.

### 3.2. MC Hyperthermia: Optimum Protocol

**Figure 4:**
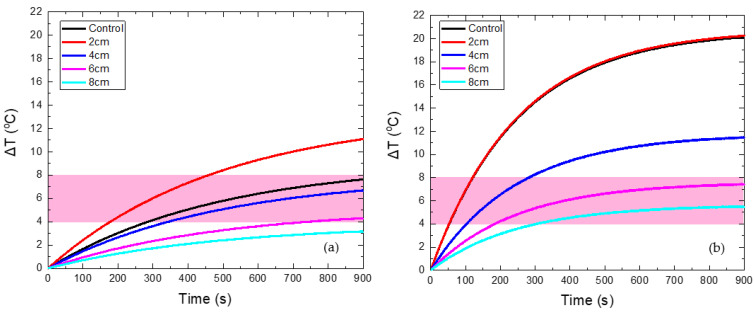
Temperature increase with time in HTP (**a**) and CTP (**b**) samples for non-moving coil, denoted as control case (black curve), and for motion under different maximum displacements (colored curves).

Four different values of maximum coil displacement x_max_ were tested at 2, 4, 6, and 8 cm. The magnetic field in the non-moving source case (control case) was set equal to 60 mT. The Δ*Τ*(*t*) curves for the HTP and CTP samples are presented in Figure 4. Figure 4a shows that for a maximum distance of 2 cm, the HTP temperature increase is much higher than the non-moving coil case (control case). Such behavior contrasted with the other cases, where the moving coil resulted in the attenuation of heating, due to eddy currents. This phenomenon can be explained from Figure S3a of the Supplementary Materials, in which it is shown that the maximum electric field reached is close to 2 cm. While the HTP is moving relatively to the coil between the two points located at x = −2 cm and x = 2 cm, and, since it oscillates, it spends more time at the extrema, where the electric field is higher compared to the other positions. As a result, eddy currents are more intense at the coil extrema and, given this, heating will be proportionally higher. For the CTP sample, the case of a maximum displacement equal to 2 cm is similar to the control, as shown in Figure 4b. This is due to the increase in EC heating, as previously explained, and also, due to the decrease of MNPs losses, when the sample is in relative motion with the center of the coil, as illustrated in Figure S3b of the Supplementary Materials.

**Figure 5:**
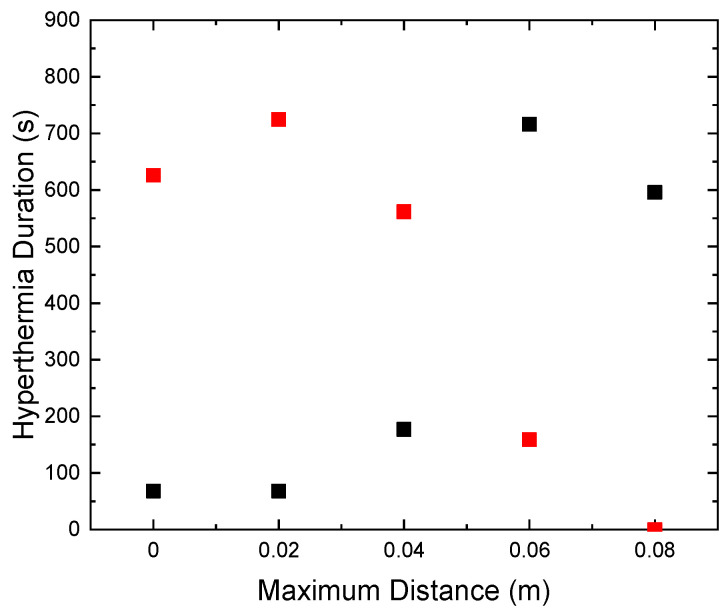
Duration time that HTP remained above 4 °C (red) and CTP (black) in MPH window (4–8 °C) for various values of maximum coil displacement.

The characteristic effective time for which the sample is retained within the MPH window is denoted here as Hyperthermia Duration. A comparison between the total time that the HTP (red points) has a temperature above 41 °C and the Hyperthermia Duration of the CTP sample (black points) is depicted in Figure 5. The desired results are those for which these two parameters get their lowest and highest value, respectively. Therefore, it clearly comes out that the maximum displacement of 8 cm is considered as the optimum one, since the HTP sample is spared—its temperature not exceeding 41 °C (Δ*Τ* < 4 °C)—and, at the same time, the CTP sample remained in the MPH window (4–8 °C) for almost 600 s.

### 3.3. PMF Hyperthermia: Optimum Protocol

**Figure 6:**
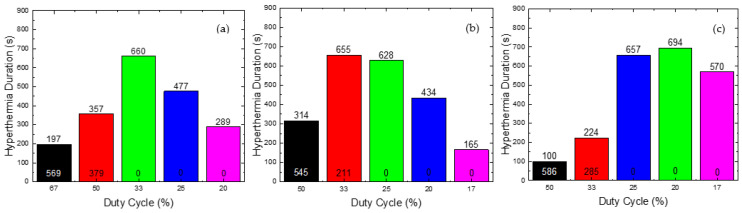
Hyperthermia Duration of CTP (written at the top of column) and HTP (at the bottom) as a function of Duty Cycle for (**a**) 45 mT, (**b**) 60 mT, and (**c**) 70 mT. The various Duty Cycles were evaluated with two criteria: (i) HTP sparing, i.e., its temperature should not exceed the value of 41 °C and (ii) the maximum Hyperthermia Duration for the CTP sample. After this evaluation, the final optimal values of Duty Cycle were found for each magnetic field used (45, 60 and 70 mT). The Duty Cycles equal to 20%, 25%, 33%, 50% and 67% are presented with purple, blue, green, red and black color respectively in (**a**,**b**). In (**c**) The Duty Cycles equal to 17%, 20%, 25%, 33% and 50% are presented with purple, green, blue, red and black color respectively.

In Figure 6, the values of this time are presented for the various Duty Cycles and AMF amplitudes used. The evaluation was done by comparing the total time that the HTP sample holds a temperature of $T_{HTP}<$ 41 °C, while the CTP sample lies in the field of MPH, 41 °C $<T_{CTP}<$ 45 °C ($\Delta {Τ}_{HTP}<$ 4 °C *και* 4 °C < Δ*T_CTP_* < 8 °C, respectively) for the various Duty Cycles tested. In order to obtain the optimum results, the first time reported should be minimized, while the second should be kept as high as possible.

As shown in Figure 6a, for a Duty Cycle of 33%, corresponding to ON/OFF = 50/100 s, the Hyperthermia Duration of the CTP is 660 s and, simultaneously, the temperature of the HTP does not exceed the value of 41 °C throughout the treatment. Therefore, those parameters were the optimum choice for the field of 45 mT. In Figure 6b, for the value of 33%, the maximum residence time of the CTP sample in the MPH window (4 °C $<\Delta {Τ}_{CTP}<$ 8 °C) is achieved, but, concurrently, there is significant heating of the HTP sample. Hence, for the AMF of 60 mT, the 25% Duty Cycle, corresponding to ON/OFF = 25/75 s, is selected. Consequently, as shown in Figure 6c, for the optimum Duty Cycle the value of 20% was selected with ON/OFF = 25/100 s. For this value, the CTP sample presented the highest Hyperthermia Duration, while the temperature increase of the HTP sample did not exceed 4 °C.

The optimum conditions are summarized in Table 1, for every AMF amplitude. The case of 70 mT, for ON/OFF = 25/100, that corresponded to a Duty of 20%, showed the better results compared to the other examined fields. More specifically, the application of this protocol resulted in maximum Hyperthermia Duration and maximum temperature increase for the CTP sample, and, at the same time, in the lowest maximum temperature for the HTP sample. Thus, this protocol was considered as the optimum one for PMF hyperthermia.

To establish the optimum PMF protocols for each magnetic field studied here (45, 60, and 70 mT), we compared the Δ*Τ*(*t*) curves obtained from classic MPH (continuously applied AMF) to those obtained from PMF hyperthermia under the optimum conditions found previously. The Δ*T* curves for the various Duty Cycles were tested and are presented in Figures S5–S10 and Figures S11–S14 of the Supplementary Materials, where the optimum operational time and Duty Cycle occurred, respectively.

**Figure 7:**
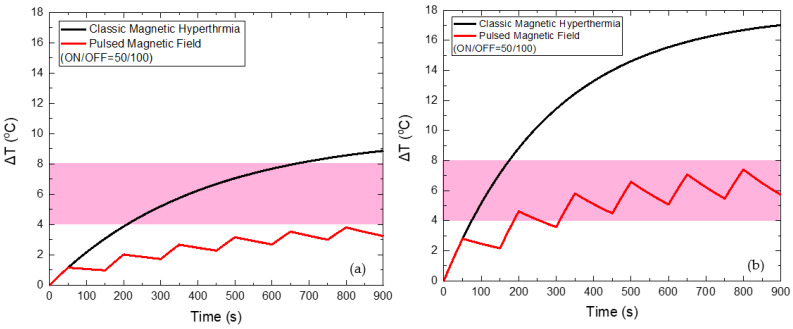
Temperature increase with time under 45 mT magnetic field after applying classic MPH (black) and PMF hyperthermia using ON/OFF = 50/100 s (red) for the HTP (**a**) and CTP (**b**) samples.

When applying a PMF of 45 mT with ON/OFF = 50/100 s, as shown in Figure 7, the reduction of the maximum temperature of HTP by 5 °C and of CTP by 9.6 °C is achieved, compared to the classic MPH, while the total Hyperthermia Duration is zero for HTP. At the same time, Hyperthermia Duration reached 660 s for the CTP sample when the corresponding time for classic MPH was only 98 s, indicating the suitability of the PMF hyperthermia method.

**Figure 8:**
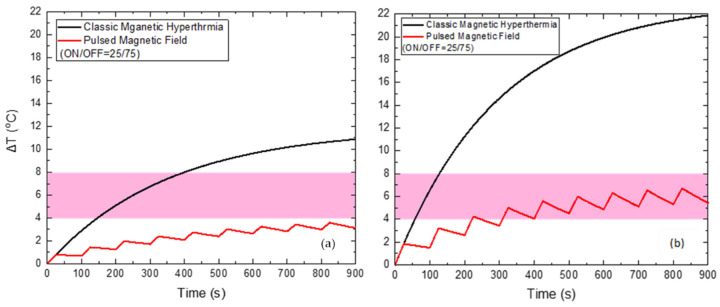
Temperature increase with time under 60 mT magnetic field after applying classic MPH (black) and PMF hyperthermia using ON/OFF = 25/75 s (red) for the HTP (**a**) and CTP (**b**) samples.

When applying a PMF of 60 mT (Figure 8) with the optimum ON/OFF = 25/75 s, the reduction of the maximum temperature of HTP by 7.3 °C and of CTP by 15.2 °C is attained, compared to the classic MPH, while the total Hyperthermia Duration is zero for HTP. At the same time, Hyperthermia Duration reached 628 s for the CTP sample, when the corresponding time for classic MPH was only 70 s.

**Figure 9:**
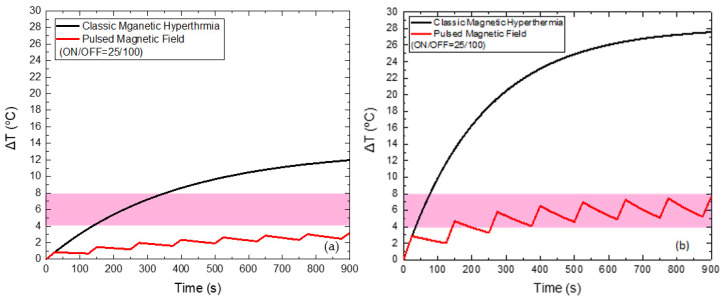
Temperature increase with time under 70 mT magnetic field after applying classic MPH (black) and PMF hyperthermia using ON/OFF = 25/100 s (red) for the HTP (**a**) and CTP (**b**) samples.

Lastly, when applying a PMF of 70 mT (Figure 9) with the optimum ON/OFF = 25/100 s, the reduction of the maximum temperature of HTP by 8.8 °C and of CTP by 20 °C is also attained, compared to the classic MPH, while the total Hyperthermia Duration is zero for HTP. At the same time, Hyperthermia Duration reached 694 s for the CTP sample, when the corresponding time for classic MPH was only 43 s.

For the AMF amplitude of 70 mT the ON/OFF sequence of 25/100 s is chosen as the optimum combination for a Duty of 25%. Since 70 mT is considered as a high field for MPH clinical application, the basic criterion is to keep the HTP sample duration as low as possible. In this way, although we exceed the limit of H × f, we succeed in minimizing the side effect in HTP—even for such a high AMF amplitude—and simultaneously maximize the damage in the CTP sample.

### 3.4. MC Hyperthermia: Validation of the Proposed Method

**Figure 10:**
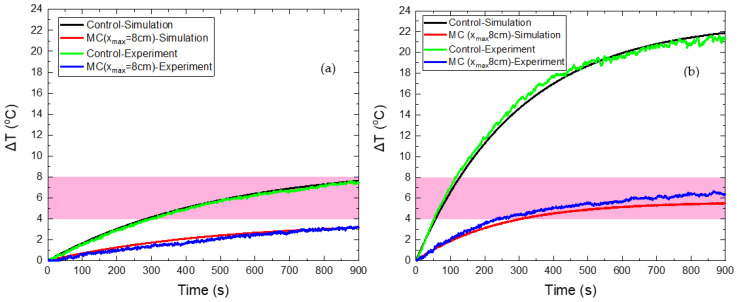
Temperature increase of HTP (**a**) and CTP (**b**) samples for a stationary coil, denoted as the control case (black color corresponds to numerical curve and green color to the experimental one) and for moving coil (red color corresponds to numerical curve and blue color to the experimental one).

In order to validate our strategy, a comparison between the numerical and the experimental results is shown in Figure 10 for both the HTP and CTP samples at the optimum displacement of coil (8 cm), as derived from the simulations.

The good agreement between the numerical and experimental data is clearly depicted in Figure 10. When the CTP sample is stable above the coil, the maximum temperature increase predicted by the numerical model was 20.1 °C, while in the experiment the corresponding Δ*Τ* reached 21.4 °C. Thus, a deviation of 6.1% is calculated between theory and experiment. In the case of relative motion, the maximum Δ*Τ* is found by the model equal to 5.6 °C, while the experimental value was 6.7 °C, resulting in a deviation of 16.4%. For the HTP sample, the maximum Δ*Τ* values calculated by the model were 7.6 °C and 3.2 °C for the fixed and moving coil, respectively, while the corresponding experimental values of Δ*Τ* were found equal to 7.5 °C (1.3%) and 3.3 °C (3%).

The obtained results indicate that the alternative protocol of MC hyperthermia resulted in the reduction of the unwanted eddy currents in healthy tissue models and, at the same time, succeeded in maintaining the beneficial effects of MNPs application in cancer tissue models, since Δ*Τ* was above the MPH limit of 4 °C. It has been shown that by tuning the parameters of coil motion, the optimal operation of MPH can be achieved, which is defined as synchronized protection and treatment efficiency. As the simulations have indicated, the spatial change of the electric and magnetic field will cause different temperature behaviors for every kind of motion that could be tested. In addition, this approach can be further explored using different mechanisms for moving the magnetic field source.

### 3.5. PMF Hyperthermia: Validation of the Proposed Method

**Figure 11:**
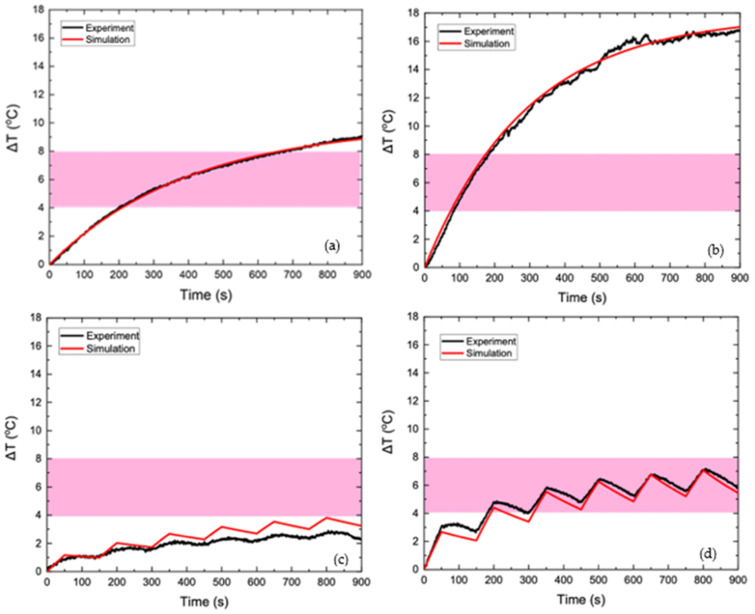
Temperature increase with time of HTP sample under 45 mT of continuously applied AMF (**a**) and 45 mT of PMF (**c**) and temperature increase with time of CTP sample under 45 mT of continuously applied AMF (**b**) and 45 mT of PMF (**d**). In all the Figures the experimental data correspond to the black colored curves, while the numerical data correspond to the red colored curves.

In order to test the reliability of the proposed strategy, the numerical results were compared to the corresponding experimental ones, as obtained in [23] after temperature measurements, by applying either a continuous AMF or a PMF of 45 mT, as shown in Figure 11.

In the HTP sample, the observed temperature increase is attributed solely to eddy currents, since this phantom did not contain MNPs. Figure 11a shows that the numerical heating curve approached the experimental data with great accuracy, which proves the validity of the model in the context of heating a phantom with eddy currents. In Figure 11b, good agreement between the simulation and experiment is also observed for the CTP sample, where heating is generated by both eddy currents and MNPs losses. As shown in Figure 11c,d, which refer to the temperature increase of the HTP and CTP samples, respectively, the values of Δ*Τ*(*t*) in the experimental process have a non-linear response to field shifts—from switched on to off and vice versa. This behavior is probably, due to the uncertainties introduced by the experimental measurement process. The measurement of the temperature through the optical fiber is performed under non-adiabatic conditions, since there is a heat exchange between the sample and the environment. The position, where the fiber is placed will affect the final result of the measurement [30]. Also, the response of the fiber to the change in temperature is not instantaneous [31] and deviates from the strictly calculated value of the temperature estimated by the algorithm. In general, the qualitative behavior of temperature in the numerical model is similar to the experiment. The goal of the development of this numerical model is to predict the temperature behavior for different parameters of the treatment, resulting in its safest and most effective use. As shown in Figure 11c, the temperature increase calculated by the numerical model for HTP is higher than that of the experiment for the entire duration of hyperthermia. In contrast, Figure 11d shows that the numerically calculated temperature of CTP is lower than the experimentally calculated one for the entire duration of hyperthermia. Therefore, the numerical model proposed can be considered as a conservative approach vis-à-vis the experiment and, thus, introduces an efficient protocol for healthy tissues sparing.

This work aims to present a versatile method for the application of safety protocols in MPH, which consists of the main clinical application. Except for hyperthermia, eddy current evolution is naturally occurring in other magnetically driven treatments, such as MRI. A typical problem in MRI clinical application is the unnecessary overheating that occurs, when large fields are applied, due to eddy current evolution, in much larger areas than the ones of interest. Therefore, diagnostic and/or therapeutic tools treatments with reliable regional control resulting in milder side effects become prerequisites, specifically in heterogenous malignancies such as cancer.

In our previous work [32], it was disclosed that eddy currents that are produced, within the MRI affect the human body. Consequently, the alternating current of the gradient coils must be carefully selected so that the resulting eddy currents do not cause any damage to the healthy tissues of the human body. Analogously, the present work unravels the beneficial role of pulsed magnetic fields and moving coils in eddy currents mitigation in an MPH setup that can be translated to an MRI setup, where the motion of the gradient and radiofrequency (RF) coils that will operate in an intermittent field (PMF) mode can combine diagnosis and therapy into the same device. From the obtained results, it is revealed that the proposed methodologies in this work make the whole treatment of MPH more effective, reducing the side effects on healthy tissues and maintaining the therapeutic efficacy of the MNPs.

## 4. Conclusions

Magnetic particle hyperthermia is a promising treatment, which, despite its many advantages over other forms of hyperthermia, suffers from the unwanted heating caused to healthy tissues by induced eddy currents. In this multiparametric work, we managed to reduce the thermal dose from the electric field to tissue-equivalent gel phantom, and, at the same time, preserve the heating to the MNPs-bearing phantom (CTP) model by introducing two alternative protocols. In the first one, a PMF was tested and, in the second, a moving heating source. Both methods were thoroughly investigated by analyzing the parameters (magnetic field, Duty Cycle, operation time, maximum displacement) that optimize the efficiency of the proposed methodology. Our method was validated with experimental results obtained under the same conditions.

When conducting MPH simulations using a PMF, even at high values of magnetic fields, such as the value of 70 mT, optimum results were obtained. The maximum temperature increase of the HTP decreased by 12 °C and the total time for which Δ*Τ* was above 4 °C decreased from 221 to 0 s. At the same time, the time during which the CTP remained in the MPH window of 41–45 °C (4 °C $<\Delta {Τ}_{\mathrm{CTP}}<$ 8 °C) increased from 43 to 694 s for MPH application of 900 s (77.1% of the total experiment). Furthermore, when looking for the optimum parameters for each field value, it was found that increasing the magnetic field requires a reduction of the Duty Cycle so that the effects of hyperthermia are the desired ones.

Similarly, notable results were recorded in the case of the moving source. For a maximum displacement of (8 cm) temperature increase, HTP decreased by 4.4 °C compared to classic hyperthermia (non-moving source) and the total time for which Δ*Τ* was above 4 °C was zero. Simultaneously, the total Hyperthermia Duration of CTP reached 600 s. In this study, it was also shown that moving the coil at short distances around the initial position of the sample, i.e., in the area, where the electric field has its maximum value, causes the results to show a contradictory trend to what is expected. The dose from the losses of the magnetic nanoparticles is reduced, while the dose from the induced electric field remains at a high level. Therefore, in this protocol of hyperthermia application it would be necessary to further study the proper movement of the coil, so that the sample is in places with low electric field values most of the time, as indicated from our FEM electromagnetic model.

Comparing the two methods in their optimum conditions (PMF hyperthermia at 70 mT with ON/OFF = 25/100 and MC hyperthermia for maximum source displacement of 8 cm), it is observed that the results of the PMF hyperthermia are slightly better. The HTP samples in both cases do not heat above the undesired temperature of 41 °C (Δ*Τ* < 4 °C), while the Hyperthermia Duration of CTP (time for which 4 °C $<\Delta {Τ}_{\mathrm{CTP}}<$ 8 °C) is longer in the case of PMF hyperthermia.

In conclusion, the two alternative protocols of magnetic particle hyperthermia that were presented are possible solutions to the already minimal side effects that dictate MPH treatment. Translating these methods to clinical practice in the future ensures the protection of healthy tissues without sacrificing the efficiency and effectiveness of the treatment provided that accurately show spatial thermometry, and that treatment duration is adjusted.

## Figures and Tables

**Table 1:** Summary of PMF hyperthermia results with the optimum ON/OFF times and Duty Cycles found for each magnetic field value (45, 60 and 70 mT).

			HTP		CTP	
Field (mT)	ON/OFF (s)	Duty	ΔΤ_max_ (°C)	Δ*t*_hyperthermia_ (s)	Δ*Τ*_max_ (°C)	Δ*t*_hyperthermia_ (s)
45	50/100	33%	3.8	0	7.4	660
60	25/75	25%	3.6	0	6.8	628
70	25/100	20%	3.2	0	7.6	694

## Data Availability

The data presented in this study are available in this article.

## Supplementary Materials

The following supporting information can be downloaded at: www.mdpi.com/article/10.3390/nano12030554/s1, Figure S1: Design of the coil-petri dish-phantom system in COMSOL; Figure S2: Spatial distribution of magnetic flux density and electric field; Table S1: Values of fitting constants; Figure S3: Radial distribution of electric and magnetic field; Figure S4: Temperature increase for 30 mT PMF; Figure S5: Temperature increase for 45 mT PMF; Figure S6: Total hyperthermia duration of CTP and HTP at 45 mT; Figure S7: Temperature increase for 60 mT PMF; Figure S8: Total hyperthermia duration of CTP and HTP at 60 mT; Figure S9: Temperature increase for 70 mT PMF; Figure S10: Total hyperthermia duration of CTP and HTP at 70 mT; Figure S11: Temperature increase under 45 mT PMF and various Duty Cycle; Figure S12: Temperature increase under 60 mT PMF and various Duty Cycle values; Figure S13: Temperature increase under 70 mT PMF andvarious Duty Cycle values; Figure S14: Temperature increase with time for PMF hyperthermia and the optimum ON/OFF values for each field; Figure S15: The magnetization versus magnetic field, M(H), dependence for the magnetite MNPs sample.
