# Supplementary material for: Numerical Simulation of Temperature Variations during the Application of Safety Protocols in Magnetic Particle Hyperthermia
Source: Nanomaterials (Basel). 2022 Feb 6;12(3):554. doi: 10.3390/nano12030554 (PMC8839068; doi:10.3390/nano12030554)
Supplement: Supplementary file 1 [file nanomaterials-12-00554-s001.zip › nanomaterials-1556876-supplementary.pdf]

nanomaterials

MDPI

Supplementary Materials

Numerical Simulation of Temperature Variations during the Application of Safety Protocols in Magnetic Particle Hyperthermia

Gerasimos Pefanis [1,2], Nikolaos Maniotis [1,2,*], Aikaterini-Rafailia Tsiapla [1,2], Antonios Makridis [1,2], Theodoros Samaras [1,2] and Mavroeidis Angelakeris [1,2]

1 School of Physics, Faculty of Sciences, Aristotle University, 54124 Thessaloniki, Greece; gpefanis98@gmail.com (G.P.); aitsiapl@physics.auth.gr (A.R.T.); anmakrid@physics.auth.gr (A.M.); theosama@physics.auth.gr (T.S.); agelaker@auth.gr (M.A.)
2 MagnaCharta, Center for Interdisciplinary Research and Innovation (CIRI-AUTH), 57001 Thessaloniki, Greece
* Correspondence: nimaniot@physics.auth.gr; Tel.: +0030-69-5511-8490

1. Fields Simulation

The spatially varied electric and magnetic fields E(x) and H(x) are calculated using the COMSOL Multiphysics [1]. After the fields have been simulated, their change along a specific axis on which the motion takes place, is estimated. Figure S1 shows the design of the coil-petri dish-phantom system that drawn within COMSOL. Due to the cylindrical symmetry of the device, the modeling takes place at the rz-plane and includes only the part where r> 0, in such a way that the rotation of this geometry around the z-axis recovers the full three-dimensional geometry. In this way the petri dish and the coil are designed in realistic dimensions in a two-dimensional model which gives us the advantage of significantly less memory space and simulation time in relation to the three-dimensional geometry.

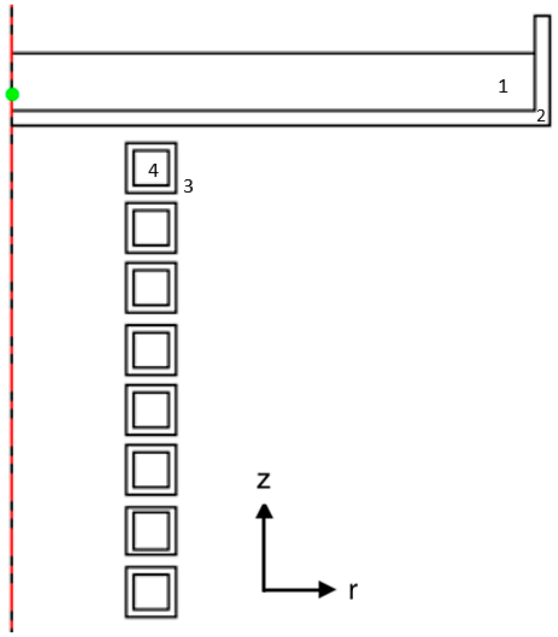

Figure S1. Design of the coil-petri dish-phantom system in COMSOL. (1) is the phantom, (2) is the petri dish, (3) are the turns of coil and (4) is the water flowing inside the coil cooling system. The green dot indicates the point at which the temperature is measured by the fiber.

In this work there was a need to find the distribution of magnetic and electric field in space. The solution of electromagnetic problem through COMSOL has been previously reported for the same conditions (f = 375 kHz, I = 160 A and current density J = 1.5x10[7] A/m[2]). The magnetic and electric field spatial distributions were obtained as the solution of the quasi-static equation for the azimuthal component of magnetic potential, $\boldsymbol{A} = A_\varphi e_\varphi$, and the characteristic material properties defined were the electric conductivity and magnetic permeability. In the time-harmonic case the equation describing the problem is:

$$(j\omega\sigma - \omega^2\varepsilon_0\varepsilon_r)A_\varphi e_\varphi + \nabla \times (\mu_0^{-1}\mu_r^{-1}\nabla \times A_\varphi e_\varphi) = J_\varphi^e e_\phi \quad \text{(S1)}$$

where ω is the radial frequency, μ_0 is the permeability of free space, μ_r is the relative permeability, σ the electric conductivity and ε_0 and ε_r the permittivity of free space and the relative permittivity. The azimuthal component of the external current density, $J_\varphi^e e_\phi$, induces an alternating current given by $\int_S J_\phi^e dS = I$, where S is the cross-sectional area of the coiled wire and I the electric current intensity, provided by the current source to the coil.

Figure S2a,b shows the obtained distribution of the magnetic and electric field in space, respectively. The electric field shows a maximum at the point, which is above the coil turns, so in this area there will be higher Eddy currents and thus higher heating as derived from equation (5) of the main manuscript.

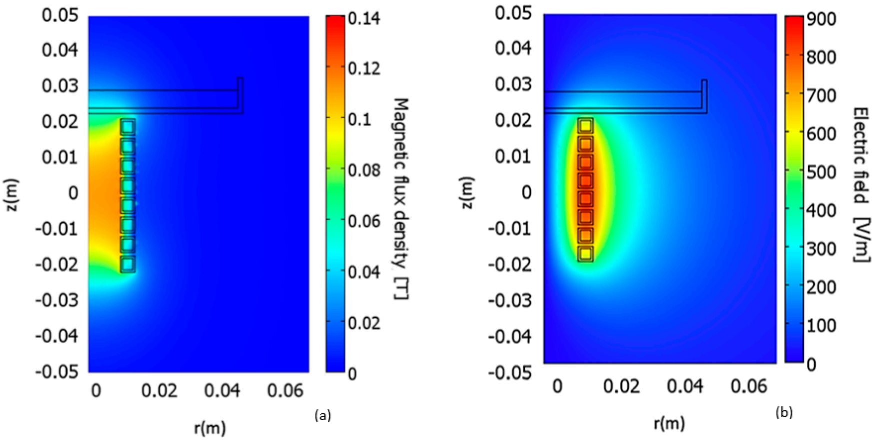

Figure S2. Spatial distribution of magnetic flux density (**a**) and electric field (**b**) obtained after FEM simulations of the experimental scheme.

In order to find the analytical expressions that describe the axial behavior of the magnetic and electric field, through COMSOL, their distribution was found along the axis movement (r-axis) of the optical fiber tip. The tip of the optical fiber is located 1mm above the bottom of the petri dish and so the axis along, which the cross-sectional distribution of H(r) and E(r) is estimated, is parallel to the bottom of the petri dish, 1mm above it and extending from 0 to 0.08 m, as shown in Figure S2a,b.

Figure S3a shows the radial distribution of the electric field on the axis of motion of the optical fiber tip for 0 m ≤r≤ 0.08 m. For the section -0.08 m ≤r≤ 0 m the distribution is symmetric, since the system has axial symmetry on the z axis. At the point r = 0m the electric field is zero, with the result that the Eddy currents are zero and therefore the heating of the model due to them, at this point, is zero. We do not expect, of course, in the experiment, that the increase in temperature measured by the optical fiber placed in the center of the HTP will be zero. The optical fiber was thought to be located just in the center of the HTP just above the center of the coil, which is experimentally impossible to achieve. Any deviation from this point results in the finite value of the electric field at the point of measurement of the optical fiber. This problem was addressed with a calibration of the utilized model. At r = 0 m, a suitable value for the electric field amplitude was given for

which the heating of the phantom in case of the non-moving coil was the same for both the experiment and the computational model. Figure S3(b) shows the radial distribution of the magnetic field, where a sharper decrease is observed in relation to the electric field. Thus, knowing the radial distribution of the electric E (r) and magnetic H (r) field, equation (7) of the main manuscript can be rewritten in the manner of the following equation:

$$r = r_{max}\sin(2\pi f_{MC} t) \tag{S2}$$

In Figure S3a,b red lines show the fitting of radial distributions. The analytical approximations found for E(r) and H(r) are described by equations (S3) and (S4), respectively. For the fittings of radial field distributions, the coefficients of determination (R^2) were 0.999 and 0.995 for the magnetic and electric field, respectively.

$$E(r) = c + \frac{q}{\sqrt{2\pi} w r} e^{\frac{-\left[\ln\frac{r}{r_0}\right]^2}{2w^2}} \tag{S3}$$

$$H(r) = q1 + (q2 - q1)\left[\frac{p}{1 + 10^{(c1-r)h1}} + \frac{1-p}{1 + 10^{(c2-r)h2}}\right] \tag{S4}$$

The fitting constants assigned to these analytical expressions along with their errors are shown in Table S1.

Fitting constant	Value	Error (±)
c	29.752	1.391
q	8.516	0.073
w	0.754	0.007
r_0	0.023	2.1×10^{-4}
q1	319,775	14.804
q2	20768.586	135.786
p	0.69	0.035
c1	0.015	3.5×10^{-5}
c2	0.025	1.6×10^{-3}
h1	−166.241	4.894
h2	−54.5	2.286

Table S1. Values of constants after fitting the electric and magnetic field distributions.

Hysteresis losses of the MNPs can be further defined as a function of the magnetic field by estimating the hysteresis loop area through the equation (S5) [2].

$$A\big(H(r)\big) = 4\alpha\mu_0 H(r) M_s \tag{S5}$$

In equation (S5) M_s is the saturation magnetization that equals to 96.3 emu/g (Figure S15) and α is the "squareness", a dimensionless parameter which shows the deviation of the hysteresis loop from a perfect square, that corresponds to oriented MNPs (α = 1), and is related to MNPs properties (size, material, degree of magnetic moments alignment to the magnetic field). For randomly oriented magnetite MNPs in the single domain regime α = 0.3 [3,4].

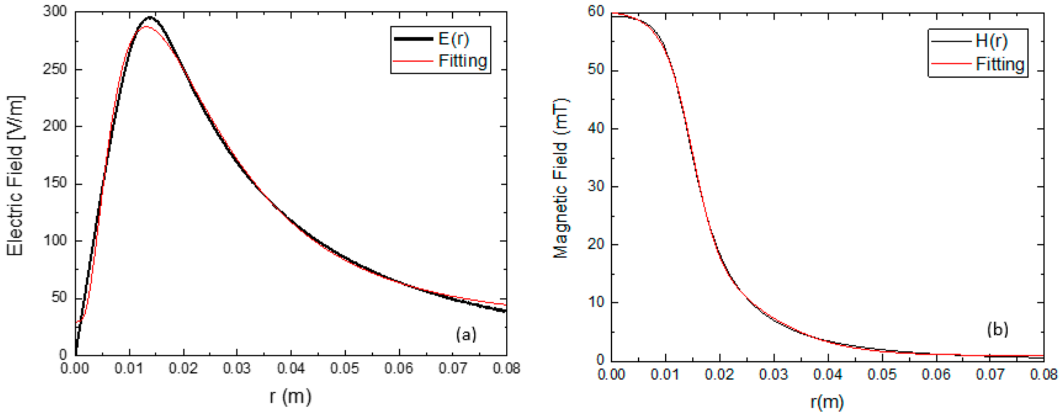

Figure S3. Radial distribution of Electric field (**a**) and magnetic field (**b**). The red line corresponds to the analytical equation with which the fitting of fields distribution was performed.

To define the heating losses as functions of the radial distance we proceed as follows: Equation (S4) is imported in Equation (S5), which in turn is imported in Equation (6) of the main manuscript, resulting in the calculation of the SAR_{MNPs}(r) for each position through which the sample passes. Also, for the Eddy current losses, equation (S3) is imported in equation (5) of the main manuscript, and we end up with SAR_{EC}(r) relation. Thus, after finding of SAR_{MNPs}(r) and SAR_{EC}(r) relationships, the code can now estimate the temperature increase ΔT(r,t) in each position.

2. PMF Hyperthermia: Optimum Operation Time

Initially, different combinations of ON and OFF times with a constant duty cycle of 33%, a value that has been already examined in a previous work [**Error! Bookmark not defined.**], were tested in order to characterize each magnetic field used, according to an optimum sequence. The evaluation was done by comparing the total time that the HTP sample holds a temperature of $T_{HTP} > 41$°C, while the CTP sample lies in the field of MPH, $41°C < T_{CTP} < 45°C$, ($\Delta T_{HTP} < 4°C$ $\kappa\alpha\iota$ $4°C < \Delta T_{CTP} < 8°C$, respectively) for the various ON/OFF combinations tested. In order to obtain the optimum results, the first time reported should be minimized, while the second should be kept as high as possible. This process was repeated for four different magnetic field values, typically used in MPH, 30, 45, 60 and 70 mT. For the value of 30 mT, the temperature of CTP did not exceed the limit of hyperthermia in any ON/OFF combination, as shown in Figure S4, and thus no further examination was performed. For all the AMF amplitudes and a duty of 33%, the ON/OFF 25/50, 50/100, 75/150 and 100/200 combinations were tested.

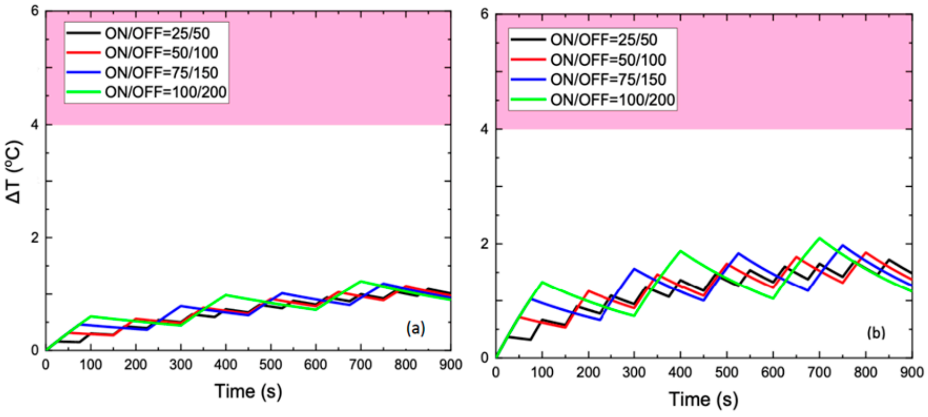

Figure S4. Temperature increase for 30 mT PMF with 33% duty for HTP (**a**) and CTP (**b**).

For 45 mT AMF amplitude, the results for HTP and CTP are shown in Figure S5, while Figure S6 shows the total hyperthermia duration (period that the phantom lies in MPH window of 41–45 °C) for CTP (at the top of column) and for HTP (at the bottom). The optimum combination, for which the CTP hyperthermia duration is quite high and simultaneously the HTP temperature did not exceed the value of 41°C, was found equal to ON/OFF = 50/100 s.

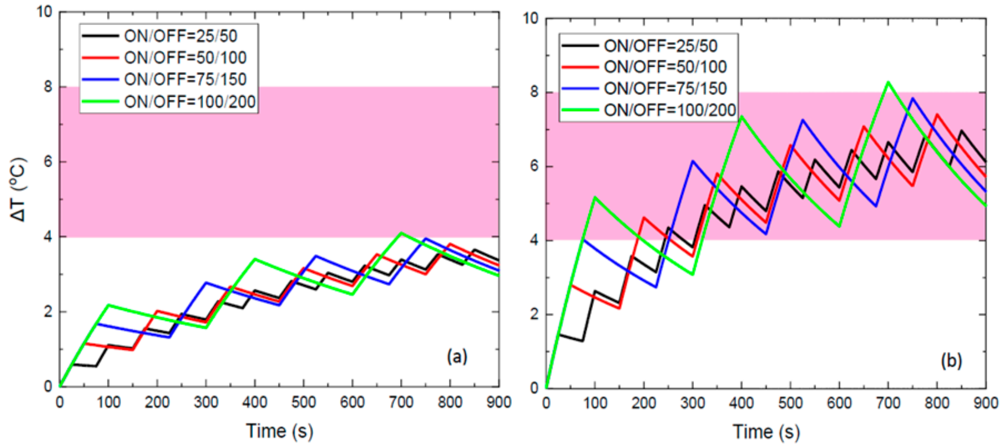

Figure S5. Temperature increase for 45 mT PMF with 33% duty for HTP (**a**) and CTP (**b**).

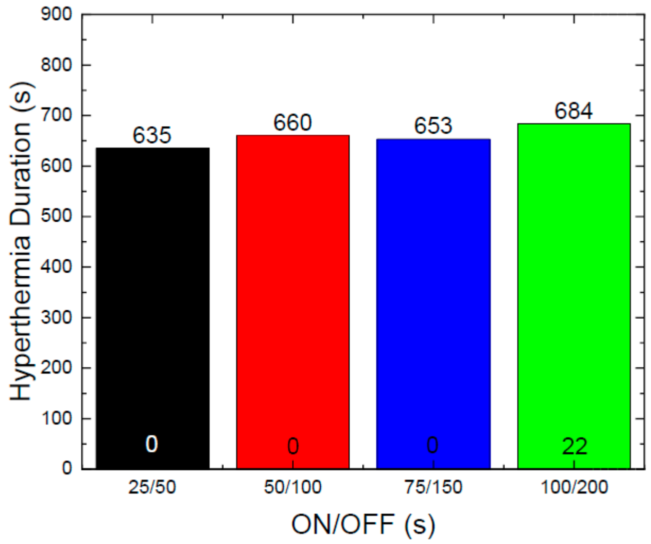

Figure S6. Total hyperthermia duration of CTP (at the top of column) and HTP (at the bottom) for 45 mT and various ON/OFF combinations.

For the 60 mT field and 33% duty cycle, the ON/OFF, 25/50, 50/100, 75/150 and 100/200 combinations were also tested. The results of ΔT(t) are presented in Figure S7, while the hyperthermia duration in Figure S8. The optimal ON/OFF, for which the hyperthermia times of CTP and HTP are the highest and the lowest, respectively, (as compared to the other ON/OFF sequence) was found equal to 25/50.

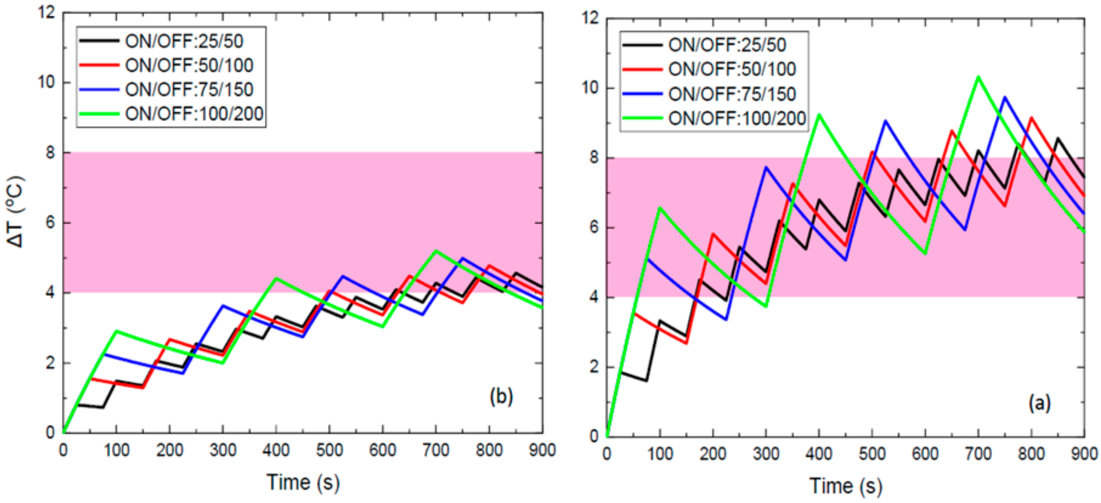

Figure S7. Temperature increase for 60 mT PMF with 33% duty for HTP (**a**) and CTP (**b**).

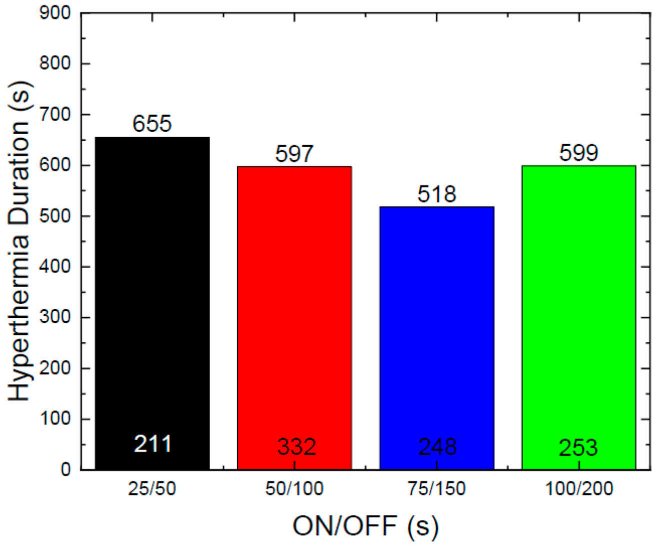

Figure S8. Total hyperthermia duration of CTP (at the top of column) and HTP (at the bottom) for 60 mT and various ON/OFF combinations.

Finally, for the AMF amplitude of 70 mT the ON/OFF sequence of 25/50 s is chosen as optimum combination for a duty of 33%. Since 70 mT is considered as a high field for MPH clinical application, the basic criterion is to keep the HTP sample duration as low as possible. With this way, although we exceed the limit of H × f, we succeed in minimizing the side effect in HTP, even for such a high AMF amplitude, and simultaneously maximizing the damage in CTP sample. The results of temperature increase and hyperthermia duration for all the studied ON/OFF sequences are shown in Figures S9 and S10, respectively.

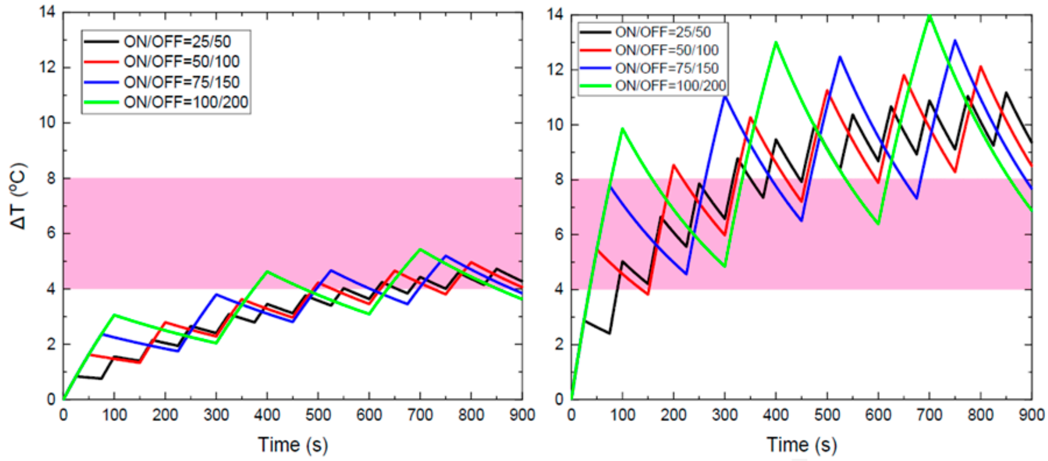

Figure S9. Temperature increase for 70 mT PMF with 33% duty for HTP (**a**) and CTP (**b**).

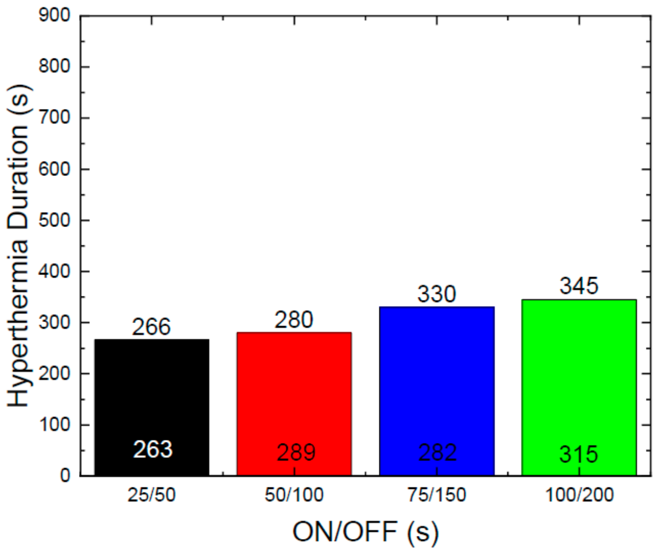

Figure S10. Total hyperthermia duration of CTP (at the top of column) and HTP (at the bottom) for 70 mT and various ON/OFF combinations.

3. PMF Hyperthermia: Optimum Duty Cycle

We then proceeded in finding the optimum duty by keeping constant the ON-time value, which is now equal to the optimum one obtained previously. Thus, changing the OFF-time value, results for different duty cycle values are obtained. The various duty cycles were evaluated again with two criteria: (i) HTP sparing, i.e. its temperature should not exceed the value of 41 °C and (ii) the maximum hyperthermia duration for the CTP sample. After this evaluation, the final optimal values of duty cycle were found for each magnetic field used (45, 60 and 70mT).

For AMF amplitude of 45 mT, the optimum operation time was 50 s, as shown in the previous section. Thus, under ON-time = 50 s, the temperature increase was estimated for various duty cycles namely 67, 50, 33, 25 and 20% corresponding to ON/OFF = 50/25, 50/50, 50/100, 50/150 and 50/200 s, as shown in the Figure S11.

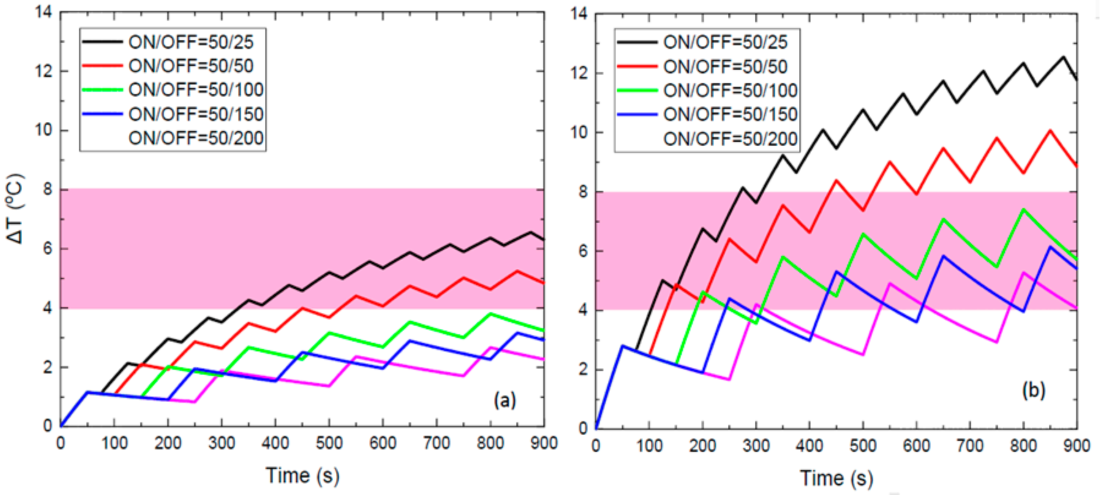

Figure S11. Temperature increase under 45 mT PMF and various duty cycle values for HTP (**a**) and CTP (**b**).

For AMF amplitude of 60 mT, the optimum operation time was 25 s. Thus, under ON-time = 25 s, the temperature increase was estimated for various duty cycles namely 50, 33, 25, 20 and 17% corresponding to ON/OFF = 25/25, 25/50, 25/75, 25/100 and 50/125 s, respectively, as shown in the Figure S12.

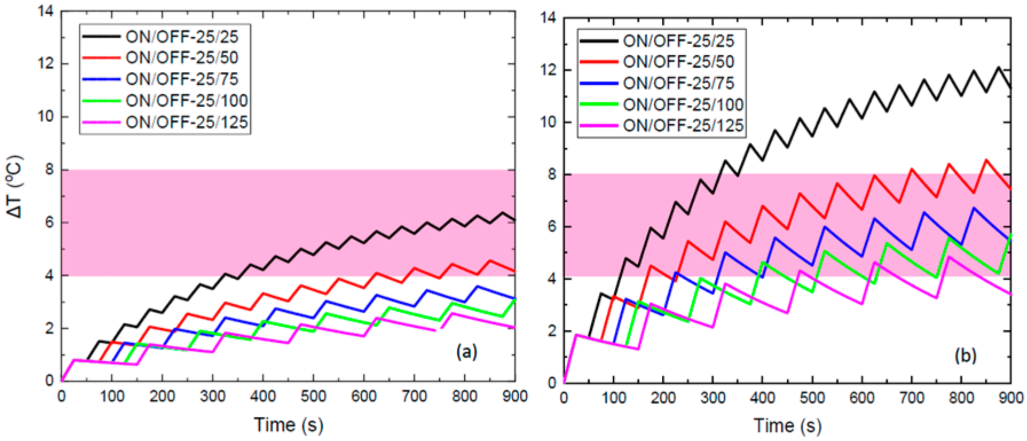

Figure S12. Temperature increase under 60 mT PMF and various duty cycle values for HTP (**a**) and CTP (**b**).

For AMF amplitude of 70 mT, the optimum operation time was 25 s and so the same duty cycles to the field of 60 mT were examined and presented in Figure S13.

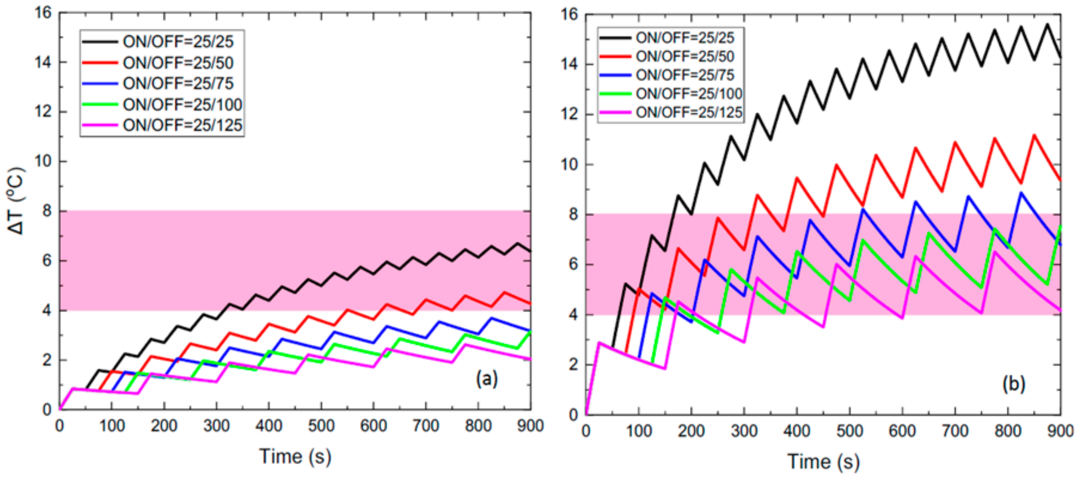

Figure S13. Temperature increase under 70 mT PMF and various duty cycle values for HTP (**a**) and CTP (**b**).

All ΔT(t) curves obtained for each magnetic field's optimum conditions, during PMF hyperthermia, are presented in Figure S14.

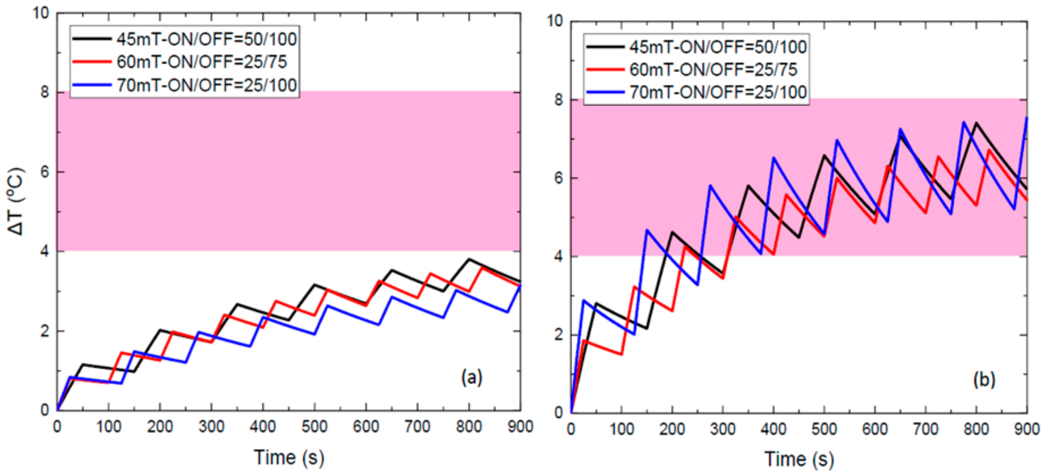

Figure S14. Temperature increase with time for PMF hyperthermia and the optimum ON/OFF values for each field, 45mT (black), 60mT (red) and 70mT (blue) in HTP (**a**) and CTP (**b**).

4. MNPs magnetic characterization

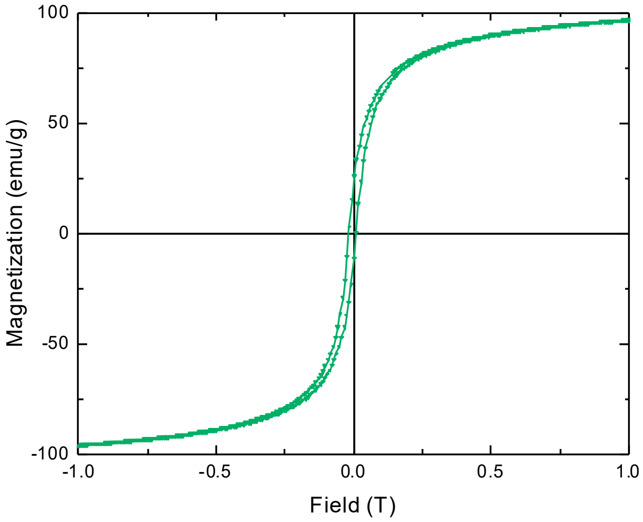

Figure S15. The magnetization versus magnetic field, M(H), dependence for the magnetite MNPs sample. The magnetization curve was obtained with a vibrating sample magnetometer (VSM) at 300 K under a static applied field of 1 T. Saturation magnetization was found equal to 96.3 emu/g.

References

1. *Comsol Multiphysics Tutorial*, version 3.5a, AC/DC Model & General Heat Model.
2. Mehdaoui, B.; Tan, R. P.; Meffre, A.; et al; Matter Mater. Phys. 2013, 102, 1–10.
3. Carrey, J.; Mehdaoui, B.; Respaud, M. Simple models for dynamic hysteresis loop calculations of magnetic single-domain nanoparticles: Application to magnetic hyperthermia optimization. *J. Appl. Phys.* **2011**, *109*, 083921. https://doi.org/10.1063/1.3551582.
4. Maniotis, N.; Nazlidis, A.; Myrovali, E.; Makridis, A.; Angelakeris, M.; Samaras, T. Estimating the effective anisotropy of ferromagnetic nanoparticles through magnetic and calorimetric simulations. *J. Appl. Phys.* **2019**, *125*, 103903. https://doi.org/10.1063/1.5082525.
5. Tsiapla, A.-R.; Kalimeri, A.-A.; Maniotis, N.; Myrovali, E.; Samaras, T.; Angelakeris, M.; Kalogirou, O. Mitigation of magnetic particle hyperthermia side effects by magnetic field controls. *Int. J. Hyperth.* **2021**, *38*, 511–522, https://doi.org/10.1080/02656736.2021.1899310.
